# Trends of fluid requirement in dengue fever and dengue haemorrhagic fever: a single centre experience in Sri Lanka

**DOI:** 10.1186/s13104-015-1085-0

**Published:** 2015-04-08

**Authors:** Senanayake AM Kularatne, Kosala GAD Weerakoon, Ruwan Munasinghe, Udaya K Ralapanawa, Manoji Pathirage

**Affiliations:** Department of Medicine, Faculty of Medicine, University of Peradeniya, Peradeniya, Sri Lanka; Department of Parasitology, Faculty of Medicine and Allied Sciences, Rajarata University of Sri Lanka, Saliyapura, Sri Lanka; Teaching Hospital, Peradeniya, Sri Lanka

**Keywords:** Dengue fever, Dengue haemorrhagic fever, Critical phase, Fluid management

## Abstract

**Background:**

Meticulous fluid management is the mainstay of treatment in dengue fever that is currently governed by consensus guidelines rather than by strong research evidence. To examine this issue we audited the fluid requirement of a cohort of adult patients with dengue fever (DF) and dengue haemorrhagic fever (DHF) in a tertiary care clinical setting.

**Results:**

This retrospective cohort study was conducted from July 2012 to January 2013 in Teaching Hospital, Peradeniya, Sri Lanka. Adult patients with confirmed dengue infection managed according to the national and WHO guidelines were included. Their fluid requirement was audited once data collection was over in both DF and DHF groups. Out of 302 patients, 209 (69%) had serological confirmation of dengue infection, comprising 62 (30%) patients gone into critical phase of DHF. Mean age of the DHF group was 30 years (range 12-63 years) and included more males (n = 42, 68%, p < 0.05). Their mean duration of fever on admission and total duration of fever were 4 days and 6 days respectively. DHF group had high incidence of vomiting, abdominal pain and flushing, lowest platelet counts and highest haematocrit values compared to DF group. In DHF group, the mean total daily requirements of fluid from 2^nd^ to 7^th^ day were 2123, 2733, 2846, 2981, 3139 and 3154 milliliters respectively to maintain a safe haematocrit value and the vital parameters. However, in DF group the fluid requirement was lowest on 3^rd^ day (2158 milliliters). DHF group had significantly high fluid requirement on 5^th^ -7^th^ day compared to DF group (p < 0.05).

**Conclusions:**

Patients in critical phase of DHF required a higher volume of fluids from the 3^rd^ day of fever and again on 5^th^ to 7^th^ day of fever. Despite being an audit, these finding could be useful in future updates of guidelines and designing research.

## Background

Dengue infection affects a large number of people mainly in tropical and subtropical regions of the world causing a significant morbidity and mortality [1,2]. Dengue is an established infection in Sri Lanka and caused major epidemics in 2002, 2004 and 2009 [3-5]. Apart from well known plasma leak, dengue infection causes multiple-organ dysfunction. The clinical spectrum ranges from mild illness to severe disease complicated by haemorrhage, fluid leakage and shock. Management of dengue does not have any specific treatment but, prompt fluid resuscitation with frequent monitoring are the main stay of life saving measures [1].

The consensus guidelines of clinical management of dengue stress on meticulous fluid management in which, fluid requirement during critical phase is calculated using many assumptions and clinical experience of experts [6]. Research evidence is scarce with regards to actual fluid requirement during critical phase of dengue infection when plasma leak happens at different rates leading to shock. Finding out of the rate and duration of plasma leak is difficult in a clinical setting, but if this information available, tailor made administration of fluid would be possible. Hence it is important to make an attempt to study/audit trends of fluid requirement in dengue infection akin to the severity of the disease. However, studies addressing this question are not available in the literature except one study that addressed the value of adequate fluid intake prior to hospitalization in dengue fever [7]. The aim of this study is to audit the fluid requirement in a cohort of patients with dengue fever (DF) and dengue haemorrhagic fever (DHF) in critical phase, and also to compare the fluid intake and many more parameters between those two groups. In addition, we sought to study the trends of these parameters over the time course of the infection.

## Results

A total of 302 patients were clinically diagnosed to have dengue infection during the study period. Of them, 209 (69%) had confirmation of the diagnosis with Dengue NS1 antigen and IgM and IgG antibodies in acute sera. Of the 209 confirmed cases, 147 (70%) were in DF group and 62 (30%) patients were in DHF group. Mean ages of the two groups were 30.2 years (range 12-62 years) and 29.7 years (range 12-63 years) respectively. The DHF group included more males (n = 42, 68%, Chi square p < 0.05) compared to DF group which had 51% (n = 75) of males. In both groups, the mean duration of fever on admission was 4 days and they had a total duration of fever for 6 days. Commonest clinical features were fever, headache, arthralgia, myalgia, nausea and flushing (Table 1). Compared to DF group, the DHF group had high incidence of vomiting (n = 39, 63%, p = 0.01**)**, abdominal pain (n = 28, 45%, p = 0.049**)** and flushing (n = 50, 81%, p = 0.028) (Table 1). Hepatomegaly, tenderness in the right iliac fossa and myocarditis were present in both groups (Table 1).

**Table 1:** **Comparison of clinical features between DF and DHF groups**

**Clinical features**	**DF group (n = 147)**		**DHF group (n = 62)**		P value*
	**n**	**%**	**n**	**%**	
Fever	147	100	62	100	-
Headache	126	86	57	92	0.167
Myalgia	130	88	57	92	0.421
Joint pain	110	75	53	85	0.063
Nausia	97	66	45	73	0.338
Vomiting	65	44	39	63	0.011
Abdominal pain	45	31	28	45	0.049
Bleeding manifestations	17	12	10	16	0.395
Flushing	98	67	50	81	0.028
Hepatomegaly	12	8	7	11	0.498
Splenomegaly	2	1	0	0	-
RIF tenderness	33	22	19	31	0.227
Pleural effusion	0	0	25	40	-
Ascitis	0	0	16	26	-
Myocarditis	4	3	4	6	0.241

*Two proportion analysis, DF – Dengue fever, DHF - Dengue haemorrhagic fever.

Change in laboratory parameters in the two groups during the ward stay were compared and are summarized in Tables 2, 3, and 4. The group DHF had significantly low platelet counts and high haematocrit values compared to DF group. Further, an early rise of leukocyte count following leucopenia was noted in the DHF group.

**Table 2:** **Comparison of haematocrit values of DF and DHF groups**

**Day from onset of fever**	**Day of defervescence**	**Haematocrit values (%)**				P value*
		**DHF group (n = 62)**		**DF group (n = 147)**		
		**Mean**	**SD**	**Mean**	**SD**	
D3	D-3	42.62	5.04	39.69	4.21	0.139
D4	D-2	41.83	5.15	39.56	5.10	0.031
D5	D-1	42.82	5.37	40.11	5.55	0.004
D6	D0	41.22	6.68	39.13	4.63	0.042
D7	D + 1	41.15	5.43	38.78	4.59	0.010
D8	D + 2	40.56	4.58	36.92	6.42	0.002
D9	D + 3	38.73	4.05	36.27	9.04	0.293

*Z test, DF – Dengue fever, DHF - Dengue haemorrhagic fever.

**Table 3:** **Comparison of platelet counts between DF and DHF groups**

**Day from onset of fever**	**Day of defervescence**	**Platelet count (x10** ^**6**^ **/l)**				P value*
		**DHF group (n = 62)**		**DF group (n = 147)**		
		**Mean**	**SD**	**Mean**	**SD**	
D3	D-3	112.5	49.5	127.9	47.6	0.244
D4	D-2	70.2	52	104.6	46.0	0.001
D5	D-1	50.8	34.8	97.4	51.9	<0.001
D6	D0	44.5	28.9	86.0	49	<0.001
D7	D + 1	45.1	33.3	79.5	44.3	<0.001
D8	D + 2	58	35.3	87.8	39.0	<0.001
D9	D + 3	93.3	45.2	111.0	46.5	0.220

*Z test, DF – Dengue fever, DHF - Dengue haemorrhagic fever.

**Table 4:** **Comparison of leukocyte counts between DF and DHF groups**

**Day from onset of fever**	**Day of defervescence**	**Leukocyte counts (x10** ^**6**^ **/l)**				P value*
		**DHF group (n = 62)**		**DF group (n = 147)**		
		**Mean**	**SD**	**Mean**	**SD**	
D3	D-3	4.40	1.6	4.03	2.01	0.436
D4	D-2	4.09	2.57	3.93	2.16	0.753
D5	D-1	4.08	2.27	4.36	4.41	0.602
D6	D0	5.68	3.55	4.47	2.68	0.029
D7	D + 1	6.08	3.57	4.73	2.56	0.019
D8	D + 2	5.68	2.73	4.88	2.02	0.111
D9	D + 3	5.62	1.96	5.45	2.30	0.791

*Z test, DF – Dengue fever, DHF - Dengue haemorrhagic fever.

**Figure 1:**
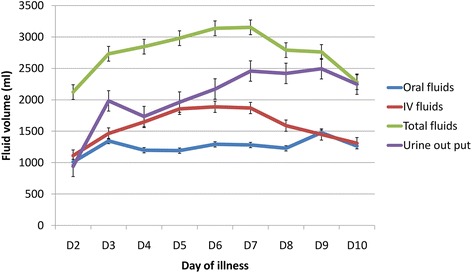
**Pattern of fluid requirement and urine output of Dengue haemorrhagic fever (DHF) group.**

**Figure 2:**
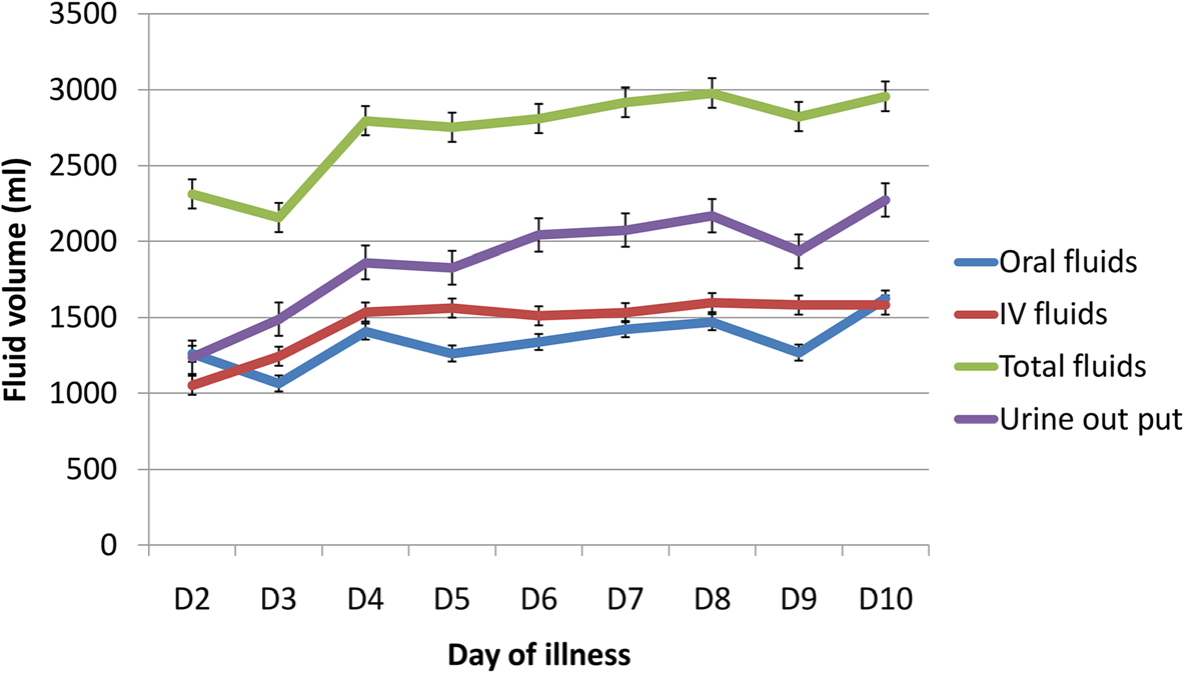
**Pattern of fluid requirement and urine output of Dengue fever (DF) group.**

**Figure 3:**
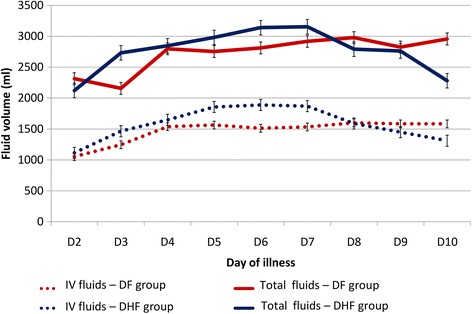
**Comparison of fluid requirement between DF and DHF groups.**

Comparison of daily fluid requirements of the two groups, both intravenous and total fluid intakes showed higher intake in DHF group from day 3 to day 7 of the illness (Table 5, Figures 1 and 2) and this difference was statistically significant from day 5 to day 7. Intravenous fluids that were administered include Normal Saline and Dextran. Normal Saline was the first line IV fluids for all, and Dextran was administered to 16 patients in the DHF group in addition to Normal Saline. The total fluid requirement in DHF group was higher on 3^rd^ day of illness (2733 ml) when DF group required the lowest intake (2158 ml). This was followed by a slight rise, plateau and a drop in trend lasting for days (Figure 3). The urine out put of the DHF group was higher from day 3 to day 9 of the illness.

**Table 5:** **Comparison of the requirements of total and IV fluid intake between DF and DHF groups**

**Day from onset of fever**	**Day of defervescence**	**Total fluid intake in ml**					**IV fluids in ml**				
		**DHF group**		**DF group**		P value*	**DHF group**		**DF group**		P value*
		**Mean**	**SD**	**Mean**	**SD**		**Mean**	**SD**	**Mean**	**SD**	
D2	D-3	2123	492	2314	1449	0.706	1113	308	1053	678	0.810
D3	D-2	2733	1457	2158	833	0.135	1464	612	1245	476	0.198
D4	D-1	2846	1250	2798	1164	0.851	1648	523	1537	460	0.290
D5	D0	2981	1247	2754	1071	0.268	1857	609	1563	531	0.004
D6	D + 1	3139	880	2812	966	0.032	1889	541	1512	417	<0.001
D7	D + 2	3154	913	2918	884	0.129	1870	555	1533	422	<0.001
D8	D + 3	2791	690	2979	936	0.255	1589	487	1599	494	0.920
D9	D + 4	2762	598	2825	762	0.782	1449	543	1583	503	0.494
D10	D + 5	2281	639	2957	1163	0.152	1310	617	1583	514	0.377

*Z test, DF – Dengue fever, DHF - Dengue haemorrhagic fever, IV – Intravenous.

## Discussion

We audited the fluid requirement of a cohort of patients with DF and DHF from the time of admission in a tertiary care hospital in Sri Lanka. Even though, fluid volume needed per day for a given patient was decided by the management team with an apparent bias, this error was minimized by use of guideline based parameters for calculation of fluid volume and doing the auditing as a separate work sometime later. Despite possible unintentional bias, we found that patients who developed DHF required a higher volume of fluids from the early stages of the illness with a higher requirement on the 3^rd^ day of fever. Moreover, DHF group needed a significantly higher requirement of fluid on 5^th^ to 7^th^ day of the illness when compared with DF group. Tenderness in the right iliac fossa was present in all patients in DHF group and they had high incidence of vomiting, abdominal pain and flushing**.** All patients of this cohort recovered fully.

In general, common clinical features observed in the whole cohort were fever, headache, arthralgia, myalgia, nausea and flushing similar to previous studies [8,9]. A higher proportion in both groups had abdominal symptoms and signs such as nausea, vomiting, abdominal pain, hepatomegaly and tenderness in the right iliac fossa. Dengue infection presenting predominantly with gastrointestinal signs and symptoms has been reported already [10,11]. We found that DHF group had high incidence of vomiting and abdominal pain compared to DF group. In the literature, severe dengue infections in adults have shown abdominal symptoms simulating acute abdomen [12].

Dengue infection causes haematological manifestations such as leucopenia with lymphocytosis and thrombocytopenia. The causative mechanisms include transient bone marrow suppression and/or binding of dengue antigens to platelets leading to antibody mediated immunological destruction of platelets. At the same time, immune mechanisms are supposed to be the causation of fluid leakage [13]. We found that DHF group had significantly low platelet counts and high haematocrit values compared to dengue fever group. High haematocrit value is a strong indicator of plasma leakage and the low platelet count is basically mediated by complex immune mechanisms in severe dengue infection [14]. It has been found that leucopenia lacks relationship to disease severity, whereas the degree of thrombocytopenia has direct association to the disease severity [14,15]. An early rise in leukocyte following critical phase and also leucocytosis with thrombocytopenia are known to occur during fluid leak [16,17].

Current management of dengue infection does not have any specific treatment except cautious monitoring and appropriate fluid replacement therapy [1]. Early diagnosis and optimal clinical management reduce the fatalities in both children and adult patients [18]. The basic pathophysiologic mechanism in severe dengue infection is the plasma leakage which occurs due to increased capillary permeability. Plasma leakage is transient and usually lasts around 24-48 hours [1]. Increased capillary permeability is caused by endothelial dysfunction due to immune mediators and by endothelial cell injury occurring in more severe disease [19-21]. Generally the plasma leakage occurs selectively into peritoneal and pleural spaces [22]. Further, it has been shown that third space fluid accumulation in severe dengue occurs in febrile phase very early, on or before the fourth day [17]. In this study, we noted that patients who developed DHF required a higher volume of fluid as early as on third day of the illness compared to uncomplicated dengue infection. Again a significant requirement of fluid was observed from 5^th^ to 7^th^ day of illness probably the critical phase. This stresses the need of initiating appropriate fluid therapy at the early stages of dengue infection without waiting until patients going in to critical phase. There is no way to predict the natural clinical course of the disease ending either DF or DHF during first two days of fever in dengue infection. The severity of the disease becomes apparent and predictable towards the latter part of the illness. When plasma leakage starts in dengue haemorrhagic fever, it may progress to shock. Prolonged shock may lead to organ hypo-perfusion resulting in progressive organ dysfunction, metabolic acidosis and disseminated intravascular coagulation which can result in severe heamorrhage [22].

This study attempted to audit the fluid requirement of DF and DHF in critical phase and hope extrapolation this knowledge would help to streamline the fluid therapy. The observed patterns of fluid therapy could be used as surrogate markers of natural fluid requirement in dengue fever. However, as a clinical audit, there may be limitation in drawing inferences due to bias. However, in a situation of lack of proper studies addressing the issue in question, this type of study would shed some light on the personal experience of dengue management. A research method of this nature may be the foundation for development of meticulous methodology to find the trends of fluid requirement in future. In this cohort of patients, mean day of fever on admission was day 4, which implies that there is a delay in hospital admission bypassing the crucial 3^rd^ day of fever when the fluid requirement is very high in DHF patients. Therefore, patients should be educated to take adequate intake of fluid to minimize the dehydration from the beginning of the illness. This implies that it would be prudent to have low threshold for hospital admission in dengue infection.

## Conclusions

We found that the trend of fluid requirement indicating a possible undetected continuous plasma leak in DHF patients from the early course of infection. Therefore, it appears that majority of dengue infections needs hospital admission by 3^rd^ day of fever to administer the required amount of fluid. If this crucial time is ignored, the disease process has a risk of progressing to severe form causing high morbidity. Preliminary evidence of this nature should be taken into account when updating the management guideline in dengue in future.

## Methods

### Ethics statement

This study was conducted with the approval from the Ethics review committee, Faculty of Medicine, University of Peradeniya, Sri Lanka.

### Study setting and population

This hospital based cohort study included subjects from July 2012 to January 2013 at the Professorial Medical Unit of the Teaching Hospital, Peradeniya, Sri Lanka where dengue case records were maintained well. The unit follows WHO criteria in clinical diagnosis that included the presence of fever and some of the following features: anorexia, headache, skin flush, generalized arthralgia, myalgia, leucopenia, thrombocytopenia and presence of warning signs of severe dengue infection [1]. All patients were managed according to the routine protocols of the unit based on national guidelines [6]. Fluid management of these patients was guided by parameters such as haematocrit, urine output, blood pressure, pulse pressure, postural drop of blood pressure and the clinical wellbeing of the patient. Both over- hydration and inadequate hydration were avoided by close monitoring of all vital signs and adjustment of fluid intake hourly. Fluid balance charts were maintained that included the kind of fluid, route of administration, urine output and other losses and fluid balance at every 12^th^ hour. During the hospital stay, each case history was closely scrutinized and categorized into either DF or DHF group. Presence of plasma leak was suggested by ultrasound examination confirmed third space fluid accumulation such as pleural effusion, ascites or raised haematocrit more than 20% of normal value. In the study cohort, comparison of the volume of fluid given over 24 hours on daily basis was calculated and compared between DF and DHF groups.

### Confirmation of the diagnosis and case definition

The disease confirmation was based on the clinical parameters and presence of dengue NS1 antigen and dengue specific IgM and IgG in acute sera, using commercial test kits (*SDBIOLINE*® Dengue IgG/M and NS1 antigen kits). Serology was performed in clinically diagnosed patients with dengue fever and blood collection for serology was done from day 6 to day 9 of the illness. Patients with clinical and laboratory evidence of plasma leakage and deteriorating cardiovascular parameters were defined as DHF group.

### Data processing and analysis

Processing was done on data gathered in the data sheets. Individual data points were stored in a computerized data base (Excel,™ Microsoft). Basic descriptive analyses were done by means of measures of central tendency. Data were analyzed using Minitab, version 14 (Minitab®) for statistical significance of the results. Two proportion Z test was used in the comparison of discrete data, where appropriate and comparison of means for continuous data were done by Z test. P value less than 0.05 was considered statistically significant.
